# An RFID Indoor Positioning Algorithm Based on Bayesian Probability and *K*-Nearest Neighbor

**DOI:** 10.3390/s17081806

**Published:** 2017-08-05

**Authors:** He Xu, Ye Ding, Peng Li, Ruchuan Wang, Yizhu Li

**Affiliations:** 1 School of Computer Science, Nanjing University of Posts and Telecommunications, Nanjing 210023, China; 1215043121@njupt.edu.cn (Y.D.); lipeng@njupt.edu.cn (P.L.); wangrc@njupt.edu.cn (R.W.); 2 Jiangsu High Technology Research Key Laboratory for Wireless Sensor Networks, Nanjing 210003, China; 3 Department of Electrical and Computer Engineering, New Jersey Institute of Technology, Newark, NJ 07102, USA; yl765@njit.edu

**Keywords:** indoor positioning, RFID, Bayesian probability, *K*-Nearest Neighbor

## Abstract

The Global Positioning System (GPS) is widely used in outdoor environmental positioning. However, GPS cannot support indoor positioning because there is no signal for positioning in an indoor environment. Nowadays, there are many situations which require indoor positioning, such as searching for a book in a library, looking for luggage in an airport, emergence navigation for fire alarms, robot location, etc. Many technologies, such as ultrasonic, sensors, Bluetooth, WiFi, magnetic field, Radio Frequency Identification (RFID), etc., are used to perform indoor positioning. Compared with other technologies, RFID used in indoor positioning is more cost and energy efficient. The Traditional RFID indoor positioning algorithm LANDMARC utilizes a Received Signal Strength (RSS) indicator to track objects. However, the RSS value is easily affected by environmental noise and other interference. In this paper, our purpose is to reduce the location fluctuation and error caused by multipath and environmental interference in LANDMARC. We propose a novel indoor positioning algorithm based on Bayesian probability and *K*-Nearest Neighbor (BKNN). The experimental results show that the Gaussian filter can filter some abnormal RSS values. The proposed BKNN algorithm has the smallest location error compared with the Gaussian-based algorithm, LANDMARC and an improved KNN algorithm. The average error in location estimation is about 15 cm using our method.

## 1. Introduction

Location-based services (LBS) are provided by a number of commercial companies, and it improves user satisfaction and offers convenience. Many LBS applications need to know the detailed location of objects. Over the years, many systems have supported automatic positioning, such as the Global Positioning System (GPS), widely used in outdoor environments all over the world. However, GPS cannot support indoor positioning because there is no signal inside buildings. In order to meet the requirements for inside buildings, many indoor positioning technologies are proposed by researchers. Ultrasonic [1], sensor [2], Bluetooth [3], WiFi [4] and magnetic field [5] technologies are used to perform indoor positioning.

Yayan Ugur et al. proposed an ultrasonic based indoor positioning system [6], which uses only ultrasonic signals and calculates the position of the mobile platform with centimeter-level accuracy. The transmitters are at fixed known positions and the time difference of arrival (TDOA) of the receiver, which is embedded on the mobile unit, is calculated to get the mobile unit’s position. The system is cost effective because it uses the existing Local Area Network of the building.

Deng Zhi-An et al. proposed a novel position transition detection algorithm [7], which allows the user heading estimation to be pervasive and suitable for daily use and for indoor pedestrian navigation using the built-in inertial sensors on a smartphone. It can be used to avoid the confusion between position transition and user turn during pedestrian walking. The results show that the proposed approach can automatically detect carrying positions with high accuracy.

Li Honggui proves that the nonlinear least square method is suitable for parameter estimation of Bluetooth signal propagation and provides the theoretical details for 3D indoor position with a Bluetooth device [8]. The results of simulation and hardware experiments show that the average of absolute precision of location estimation is at meter level.

Han-Sol Kim et al. proposed an indoor positioning system [9] with a particle filter system where the weights of particles are updated by multiple magnetic sensors and three magnetic field maps: a horizontal intensity map, a vertical intensity map and a direction information map. The system integrates magnetic field map navigation and an encoder system. The results show that the estimated position of the proposed system is more accurate than that of previous systems.

Nascimento Hitalo J.B. et al. proposed a positioning algorithm [10] based on Bayes inference to locate objects in 3D WLAN indoor environments. This is a fingerprint technique and the average positioning error is about three meters.

With the increasing number of mobile robots controlled by a mobile device, Chao Chun-Tang et al. proposed a visual control interface for a mobile robot with a single camera to estimate the 3D position of a target [11]. WiFi is used to transmit the control signal and video from the mobile robot. The presented 3D estimation method is based on triangulation.

Radio frequency identification (RFID) [12] is the key technology necessary to realize the Internet of Things (IoT) and cyber-physical systems (CPS), widely used in health monitors [13,14], smart homes [15], smart cities [16], vehicle location [17], construction [18], supply chain management [19] and object tracking [20], etc. RFID has the advantages of low cost, long life, low power consumption and easy deployment, which attracts many researchers to use it in indoor environments [21,22,23]. Compared with ultrasonic, WiFi and Bluetooth, RFID used in indoor positioning is more cost and energy efficient. The typical RFID systems consists of tags, readers and a back-end computer system. There are two types of tags: active and passive, where the active tag has a battery and can send the information at hundreds of meters, and the passive tag depends on the emitted energy of the reader’s antenna instead of using a battery to transmit information. There are four different RFID frequency bands [24]: low frequency (LF)—125~134 kHz, high frequency (HF)—13.56 MHz, ultra-high frequency (UHF)—433~956 MHz and microwave frequency (MF)—2.45 GHz. Ni Lionel M. et al. first used active RFID with the concept of reference tags for indoor location sensing [25], called LANDMARC, and as they used K-nearest reference tags’ coordinates to locate unknown tags, it is also called a *K*-Nearest Neighbor (KNN) algorithm [26]. Benelli Giuliano et al. uses LF RFID to track different typologies of pebbles on beaches [27]. Mi Jian et al. designed an HF-band RFID system with multiple readers and passive tags for indoor mobile robot self-localization [28]. Own Chung-Ming et al. uses UHF RFID technologies for real-time bus recognition in the Taipei bus station [29], where the active RFID tag on the bus is combined with the infrared receiver. Hsu Chien-Chang et al. proposed a sensor-assisted RFID-based indoor tracking system [23] which uses active RFID technology to identify the location of elderly people living alone. The active RFID system may exhaust much more energy than the passive RFID system. The LF RFID system has a short reading range at centimeter-level and is not suitable for large-scale location usage.

There are two types of Received Signal Strength indicator (RSS, many references also refer to it as RSSI) based indoor positioning algorithms in RFID systems: triangulation and reference tags. The triangulation method uses the geometry of circles, spheres or triangles to determine the locations of the target by estimating distances. The following methods are usually used to estimate the distance in triangulation based position systems: Time of Arrival (TOA) [30] and Angle of Arrival (AOA) [31]. The authors [32] present a novel passive RFID localization algorithm based on elliptical trilateration in smart home environments, where they obtained an average error of 16.08 cm for all objects. The reference tags method first requires the building of a database of known RSS signals of the RFID tags’ locations. It then uses a positioning algorithm to calculate the target tag’s location. In the literature [33], the authors present a maximum intersected tags method to estimate the target’s location. The results show that the location error is about 30~60 cm. Table 1 gives the comparisons of different positioning methods. The COTS reader is a commercial off-the-shelf reader.

Due to the low cost and easy deployment of passive UHF RFID it is a widely used and researched positioning method. Buffi, A. et al. proposed a phase-based technique for localization and tracking of UHF RFID tags moving along a conveyor belt [34], and the numerical results show that a centimeter-level accuracy in the tag position estimation can be achieved even in a rich multipath environment. In order to overcome the limitations of current RFID systems, Guidi, F. et al. proposed the joint adoption of RFID and ultra-wideband (UWB) tag technologies [35,36], which are introduced to reduce the positioning error thanks to the large bandwidth involved. Arnitz D. et al. give a proof of concept for UWB-based ranging in passive UHF RFID [37], which is robust to multipath propagation. UWB RFID will offer high accuracy localization capabilities in next generation RFID systems. However, the UWB RFID is limited in the range of reader-tag communication [38] and it is costly, as the high accuracy localization requires three readers. To overcome such limitations, Guidi, F. et al. investigated the possibility of jointly adopting millimeter-wave (mmW) technology with passive RFID [39], and the results show that the proposed scheme can outperform the UWB one as it requires a lower number of pulses to achieve the same reader-tag distance and consequently a reduced complexity.

LANDMARC [25] is the first method to use reference tags to locate the target, and its positioning accuracy is about 72 cm. The KNN algorithm [25] is a kind of references tag positioning method, mainly used to estimate the location according to the measured RSS compared with the known RSS value recorded in the database. The KNN is widely used and improved in indoor environment positioning. Li D. in literature [40] proposed a feature-scaling-based k-nearest neighbor (FS-kNN) algorithm for achieving improved localization accuracy, while FS-kNN can achieve an average location error as low as 1.70 m in WiFi fingerprint-based indoor positioning systems. An improved KNN algorithm was introduced by Liang X. et al. which is beneficial to location estimation in a real GSM network [41]. In order to meet the demand of localization precision, LANDMARC is improved as well. In order to find the *k* value which leads to the best accuracy in each environment, an adaptive KNN algorithm is proposed for RFID location systems [42]. Because the multipath effect and the various indoor environments significantly affect the positioning accuracy, the overall accuracy of LANDMARC fluctuates. In order to solve this problem, Liu X. et al. proposed LANDMARC with an improved KNN algorithm for RFID location systems whose location accuracy is increased by 10.1% on average [43].

This paper mainly focuses on the passive UHF RFID-based indoor positioning method. Inspired by the WiFi indoor positioning algorithm [10] and the KNN algorithm [26], and inspired by Sun Y. and Gu F. who use the sparse signal to descript all the information of the original signal with high probability in the literature [44], we propose an RFID indoor positioning algorithm based on Bayesian probability and *K*-Nearest Neighbor, using a Gaussian filter to filter the abnormal Received Signal Strength (RSS) value and using the proper k value and the Bayesian estimation method to improve the accuracy of the location. Rather than WiFi technology [10] and active RFID technology [25], we use passive UHF RFID technology to perform indoor positioning resulting in a lower location error of about 15 cm. When comparing the proposed algorithm with the traditional LANDMARC algorithm and other related algorithms, the results show that our algorithm performs with a higher location accuracy.

The rest of the paper is organized as follows: In Section 2, we examine the RFID signal characteristic of RSS and phase. Section 3 gives the Bayesian theorem used in our algorithm. In Section 4, an RFID indoor positioning algorithm is proposed where a Gaussian filter is used to filter abnormal RSS values and Bayesian estimation is used to improve positioning accuracy. Section 5 presents the experiments. Finally, Section 6 concludes the paper.

## 2. RFID Signal Characteristic

In the following, we use the commodity off-the-shelf RFID reader—Impinj R420 RFID reader and EPC Gen2 UHF RFID tags to perform the test for the signal characteristic of RSS and phase.

### 2.1. RSS

**Figure 1:**
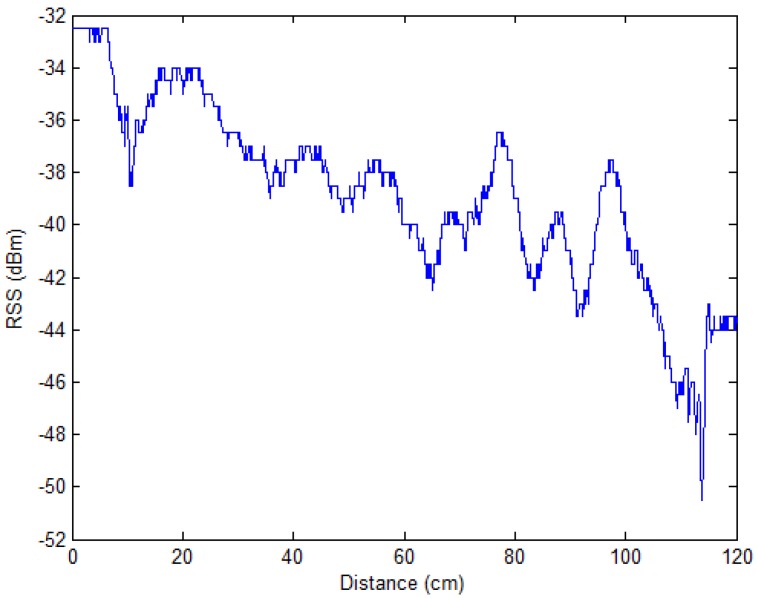
The RSS value changes according to distance.

RSS is an important eigenvalue of RFID signals, which can be obtained by the RFID reader reading the RFID tags. Through testing, we found that the RSS value has a linear relationship with the distance between the antenna of the RFID reader and the RFID tags. In Figure 1, we do the test with a continuously increasing distance from 0 to 120 cm by slowly moving an electric toy car equipped with an RFID tag further away, step by step. At the same time, the RFID reader reads the tag and records the RSS value. When the tag is far enough away from the reader’s antenna, the RSS value does not change significantly. From Figure 1, we can see that when the distance is at 120 cm, the RSS value is stable.

**Figure 2:**
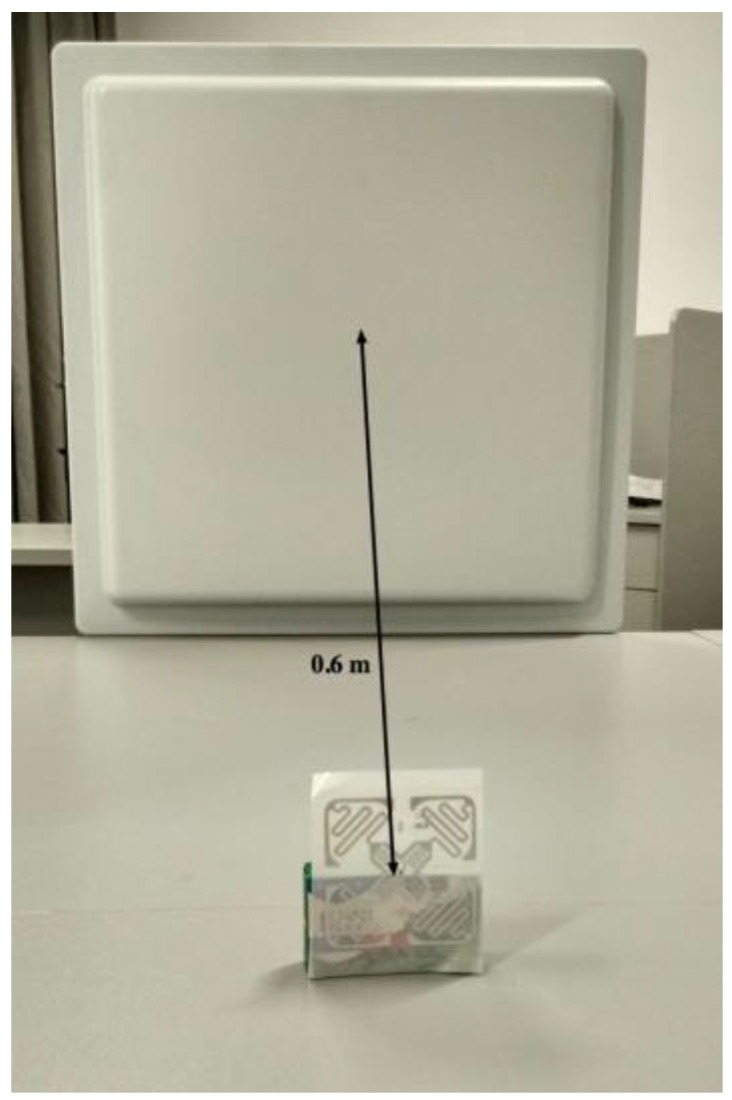
Obtaining the RSS value.

**Figure 3:**
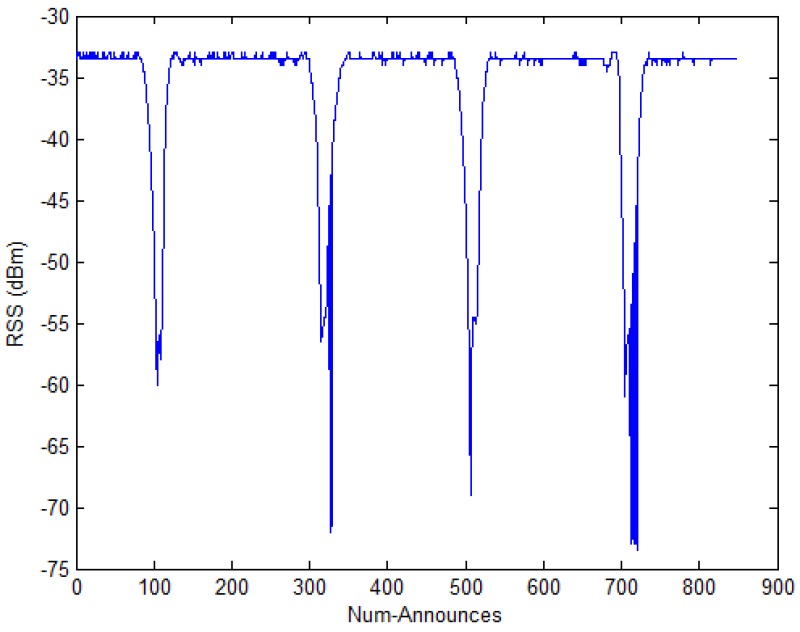
The RSS value in an interference environment.

In addition, we put the tag approximately 0.6 m in front of the reader’s antenna, as shown in Figure 2. At the same time, we recorded the RSS value. When there are shelters to pass through between the reader and the tag, the RSS value fluctuates greatly, which is shown in Figure 3. Num-Announces are the cumulative sums of numbers of a tag read successfully by the RFID reader, which is normally at a rate of approximately 40 readings per minute. From Figure 3, we can see that the fluctuations are generated when the Num-Announces are about 100, 320, 500 and 715, respectively. This is because we repeatedly put a hand in the space between the reader and the tag, causing interference with the wireless communication which influences the measured RSS.

### 2.2. Phase

**Figure 4:**
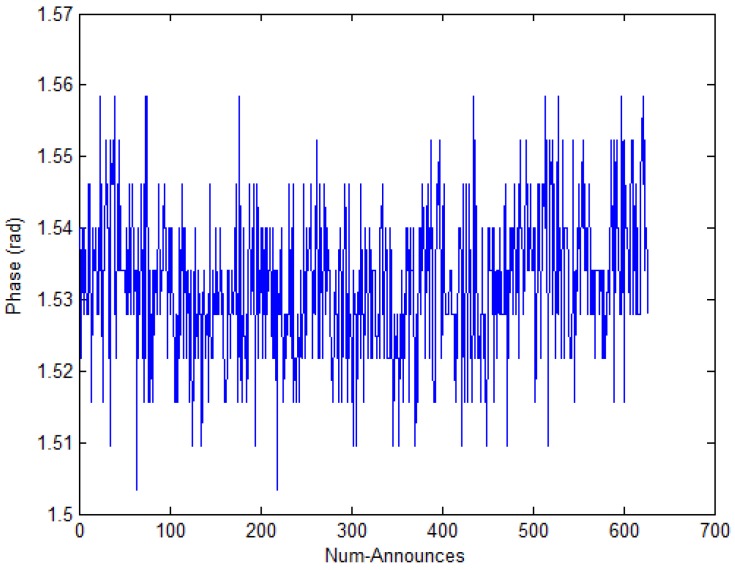
The phase value is stable in the fixed distance.

The phase is another important eigenvalue of RFID signals which the RFID reader antenna can read from the tag [45]. Similarly, we place the tag at 0.6 m as shown in Figure 2. At the same time, we record the phase value. As Figure 4 shows, if the distance between the tag and the reader is fixed, the phase value changes from 1.50 to 1.56 and the mean of the phase is 1.53, which is caused by the multipath effect and the various noises in the indoor environment. However, we can consider 1.53 as the phase value in the experiment through simple data processing. We hold the view that this characteristic of phase in a fixed distance belongs to a stable status.

**Figure 5:**
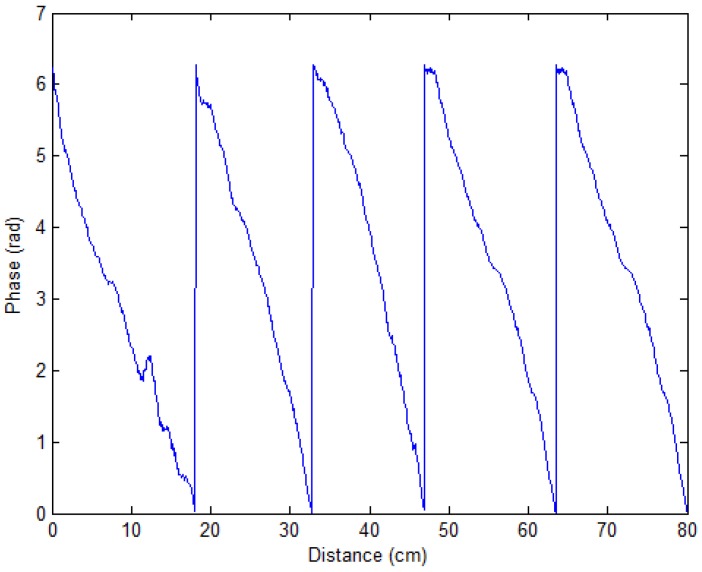
The phase value is cyclical and linear with changeable distance.

We put the RFID tag in front of the antenna of the RFID reader and kept away from it while we used the RFID reader to read the phase value at the same time. As shown in Figure 5, we found that the phase value has a linear relationship with the distance and is cyclical. In addition, the phase difference [46] of tag sensitivity makes it hard to use passive RFID for large-scale location usage.

Thus, if we use the RFID signal to carry out indoor positioning, we must overcome the noise in indoor environment and the cycle characteristic of the phase. In this paper, we mainly solve the noise problem which influences the location accuracy. In the following, we will use a Gaussian filter to filter abnormal RSS values and use Bayesian estimation with the KNN algorithm to improve positioning accuracy in RFID systems.

## 3. Bayes Theorem

The formula for conditional probability in statistics is:

(1) P(AB)=P(A)P(B|A)=P(B)P(A|B),

That is, the probability P(AB) that event *A* and event *B* occur at the same time is equal to the probability P(A) that event *A* occurs multiplied by the probability P(B|A) that event *B* occurs under the probability of the occurrence of event *A*. Or the probability P(AB) is equal to the probability P(B) that event *B* occurs multiplied by the probability P(A|B) that event *A* occurs under the probability of the occurrence of event *B*. From Equation (1), we can get:

(2) $$P\left(B|A\right)=\frac{P\left(B\right)P(A|B)}{P\left(A\right)}$$

Assume that event *B* is a probability space which is composed of independent events, then P(A) can be expanded by the full probability formula:

(3) $$P\left(A\right)=P\left(A|B_{1}\right)P\left(B_{1}\right)+P\left(A|B_{2}\right)P\left(B_{2}\right)+\cdots +P\left(A|B_{n}\right)P\left(B_{n}\right)$$

Thus, the Bayesian formula can be expressed as:

(4) $$P\left(B_{i}|A\right)=\frac{P\left(A|B_{i}\right)P\left(B_{i}\right)}{P\left(A|B_{1}\right)P\left(B_{1}\right)+P\left(A|B_{2}\right)P\left(B_{2}\right)+\cdots +P\left(A|B_{n}\right)P\left(B_{n}\right)}$$

$P\left(B_{i}|A\right)$ refers to the posterior probability, but $P\left(A|B_{n}\right)P\left(B_{n}\right)$ refers to the prior probability, and $P\left(B_{i}\right)$ refers to the basis probability. The Bayesian formula also can be expressed as:

(5) $$P\left(B_{i}|A\right)=\frac{P\left(A|B_{i}\right)P\left(B_{i}\right)}{\sum_{i=1}^{n}P\left(B_{i}\right)P\left(A|B_{i}\right)}$$

Normally, P(A|B) is not equal to P(B|A). However, the two have a definite relationship, where Bayesian law is the statement of this relationship. Bayesian law is the relationship of the conditional probability and the marginal probability of random events *A* and *B*, which is shown in the following formula:

(6) $$P\left(A|B\right)=\frac{P\left(B|A\right)P\left(A\right)}{P\left(B\right)}\propto L\left(A|B\right)P\left(A\right)$$

Where P(A|B) is the possibility of event *A* occuring in the case of event *B* having occurred, P(A) is the prior probability (also called the marginal probability) of event *A* which is unaffected by any factor of event *B*, P(A|B) is the conditional probability of event *B* having already occurred and is also called the posterior probability of event *A*, P(B|A) is the conditional probability of event *A* having already occurred and is also called the posterior probability of event *B*, and P(B) is the prior probability of event *B* which is unaffected by any factor of event *A*. In addition, sometimes $\frac{P\left(B|A\right)}{P\left(B\right)}$ is also called the standardized likelihood. Then the Bayesian law can be expressed as: Posterior probability = Standardized likelihood × Prior probability.

In summary, the parameters are being estimated as a random variable which is a specific distribution form, and the probability density distribution f(x|θ) is transferred as the posterior probability h(x|θ). It first obtains the probability density distribution and then obtains the parameter estimation value through mathematical expectations. The specific steps of the Bayesian estimate are used in our method and listed as follows:

(1) Confirm the prior distribution π(θ) of parameter θ.
(2) Obtain the joint probability density distribution f(x|θ) through joint probability density distribution $x=\left(x_{1},x_{2},x_{3},\cdots ,x_{n}\right)$.
(3) Obtain the posterior probability h(x|θ) of parameter θ.
(4) Obtain the estimated value $\hat{\theta }$

## 4. Proposed Positioning Algorithm

The indoor environment is changeable because of the intensity of the population, the random walking behavior of person, the path’s effect and so on. This will make the RSS value of the RFID tag fluctuate around a certain value. The RSS value in the same location can be approximated by the Gaussian probability distribution, and the probability density function can be expressed as the following:

(7) $$P\left(x\right)=\frac{1}{\sigma \sqrt{2\pi }}e^{-\frac{{\left(x-\mu \right)}^{2}}{2\sigma^{2}}}$$

where x represents the actual RSS value, $\mu =\frac{1}{m}\sum_{i=1}^{m}RSSI_{i}$ represents the mean of the RSS value, $\sigma =\sqrt{\frac{1}{m-1}\sum_{i=1}^{m}{\left(RSSI_{i}-\mu \right)}^{2}}$ and σ represents the standard deviation.

In order to overcome the noise in the indoor environment, we used a Gaussian filter to filter the abnormal RSS value and used Bayesian estimation to improve positioning accuracy. The proposed positioning algorithm based on Bayesian probability and KNN (BKNN) has the following three processes: (1) KNN to select k reference tags, (2) the Gaussian filter process and (3) the Bayesian estimation process. The details of algorithm BKNN are as follows:

• KNN

Suppose there are n readers and m reference tags in an RFID systems, and u target tags are need to be positioned. The RSS vector of the target tags is $s=\left(s_{1},s_{2},s_{3},\cdots ,s_{n}\right)$, which indicates the RSS value of the target tags detected by the reader i, i∈(1,n). For the reference tags, the corresponding RSS vector is $\theta =\left(\theta_{1},\theta_{2},\theta_{3},\cdots ,\theta_{n}\right)$. The Euclidean distance is defined as $E_{j}=\sqrt{\theta_{i}-s_{i}}$, j∈(1,m), which is based on the target tag and reference tag. The Euclidean distance E is used to measure the proximity of the target tag and the reference tag, that is, while the reference tag is close to the target tag the E value should be smaller. Therefore, when there are m reference tags, the E value of the target tag p and all reference tags should be smaller. Accordingly, the E value vector of the target tag p and all reference tags can be expressed as $E=\left(E_{1},E_{2},E_{3},\cdots ,E_{m}\right)$, and sorted in order from small to large which is recorded as $E^{\prime }=\left(E_{1}{}^{\prime },E_{2}{}^{\prime },E_{3}{}^{\prime },\cdots ,E_{m}{}^{\prime }\right)$. From this sequence, the k reference tags are selected and the weighted average value is calculated according to the known tag position, and the approximate position of the target tag can be estimated.

• Gaussian Filter Process

In order to make the collected RSS value close to the real value and thus improve the positioning accuracy, we need to filter the fluctuating RSS value which is generated by the probability event or noise interference. Take the RSS collection process as a normal distribution model. When we calculate the RSS, assuming that it is a Gaussian distribution in which μ represents the mean of the RSS value and σ represents the standard deviation. The result of the density function of x is expressed as the following:

(8) $$f\left(x\right)=\frac{1}{\sigma \sqrt{2\pi }}e^{-\frac{{\left(x-\mu \right)}^{2}}{2\sigma^{2}}}$$

where $\mu =\frac{1}{m}\sum_{i=1}^{m}RSSI_{i}$, $\sigma =\sqrt{\frac{1}{m-1}\sum_{i=1}^{m}{\left(RSSI_{i}-\mu \right)}^{2}}$, $RSSI_{i}$ is the *i*-th RSS value, and m is the measurement time. The greater the value of σ is, the better the smoothing effect of the Gaussian filter is. We select the a probability is greater than 0.6 which is in the high probability generation area, that is 0.6≤f(x)≤1, 0.15σ+μ≤x≤3.09σ+μ. This range of RSS values is selected, and then the geometric average is calculated which is regarded as the target tag’s RSS value.

• Bayesian Estimation Process

When calculating the target tag’s coordinate using the LANDMARC algorithm, a different k value will produce a different coordinate. The measurement can be repeated *k* times. Suppose the measurement is independent, according to Bayesian theory, the prior probability model and the posteriori probability model can be set up. The prior probability model is used to estimate the probability at *k* time of the target’s probable position at *k* − 1 time. After that, the posteriori probability model is set up according to the measurement value of the target’s position at *k* time, and then the final position can be calculated. The details are shown in the following.

Let $Z_{k}=\left\{Z_{i},i=1,2,3,\ldots ,k\right\}$ represents the sets of all measured *k* tags’ coordinates, and $Z_{i}$ is the *i*-th tag’s measured coordinates. $p\left(Z_{k}|X_{k}\right)$ is the probability of the unknown tag at $X_{k}$ position under the known set $Z_{k}$, and $p\left(Z_{k}|X_{k}\right)$ is also called as posteriori probability; $p\left(X_{k}|Z_{k-1}\right)$ is the prior probability which represents the position estimation of unknown tag under the unknown set $Z_{k}$. Assuming that the measured values are independent of each other, we can get the following formula according to Bayesian formula:

(9) $$p\left(X_{k}|Z_{k}\right)\propto p\left(Z_{k}|X_{k}\right)\times p\left(X_{k}|Z_{k-1}\right)$$

The proposed Bayesian estimation based positioning algorithm is as follows:

(1) Determine the prior probability: $p\left(X_{k}|Z_{k-1}\right)$. Suppose $X_{k}=\left(x,y\right)$, $X_{k-1}=\left(x_{k-1},y_{k-1}\right)$, we can obtain the following formula:
  (10) $$p\left(X_{k}|Z_{k-1}\right)=\frac{1}{\sigma_{1}\sqrt{2\pi }}e^{-\frac{D_{1}^{2}}{2\sigma_{1}^{2}}}$$
  where $D_{1}$ is the distance between $X_{k}$ and $X_{k-1}$.
(2) Determine the posteriori probability: $p\left(Z_{k}|X_{k}\right)$, which is shown in the following formula.
  (11) $$p\left(Z_{k}|X_{k}\right)=\frac{1}{\sigma_{2}\sqrt{2\pi }}e^{-\frac{{\left(D_{2}-Q\right)}^{2}}{2\sigma_{2}^{2}}}$$
  where $p\left(Z_{k}|X_{k}\right)$ is the probability of the unknown tag at $X_{k}$ position and the measured distance is $D_{2}$, Q represents the distance between the unknown tag and the reader’s antenna, and $\sigma_{2}$ represents the uncertainty variance of the measured distance. In our algorithm, four reference tags around the target tags’ position are selected, where these positions are $A\left(a_{1},b_{1}\right),B\left(a_{2},b_{2}\right),C\left(a_{3},b_{3}\right),D\left(a_{4},b_{4}\right)$, then we can get the value of $D_{2}=\sqrt{{\left(x-a_{i}\right)}^{2}+{\left(y-b_{i}\right)}^{2}}$.
(3) Calculate the probability distribution function of the unknown tag’s position. According to Formulas (9)–(11), we can calculate the $p\left(X_{k}|Z_{k}\right)$ which represents the probability distribution function of the target tag’s location. We then get the following formula:
  (12) $$p\left(x,y\right)=\overset{4}{\underset{i=1}{\prod }}p_{i}\left(X_{k}|Z_{k}\right)=C{\left(\frac{1}{\sigma_{1}\sigma_{2}\sqrt{2\pi }}\right)}^{4}e^{f\left(x,y\right)}$$
  where
  (13) $$f\left(x,y\right)=\overset{4}{\underset{i=1}{\sum }}f_{i}\left(x,y\right)=-\frac{1}{2}\overset{4}{\underset{i=1}{\sum }}\left\{\frac{1}{\sigma_{1}^{2}}\left[{\left(x-x_{k-1}\right)}^{2}+{\left(y-y_{k-1}\right)}^{2}\right]+\frac{1}{\sigma_{2}^{2}}{\left(\sqrt{{\left(x-a_{i}\right)}^{2}+{\left(y-b_{i}\right)}^{2}}-q_{i}\right)}^{2}\right\}$$

In Equation (13), $q_{i}$ is the distance between the reference tag and the target tag. The value of (x,y) which could make p(x,y) to be the maximum value can be regarded as the target position. The exponential function is monotonic, and we can find the (x,y) that can make f(x,y) has the maximum. The target function f(x,y) contains nonlinear factors, we define $g_{i}\left(x,y\right)=\sqrt{{\left(x-a_{i}\right)}^{2}+{\left(y-b_{i}\right)}^{2}}$ and expand the approximate value ($x^{\prime }$, $y^{\prime }$) with Taylor expansion type, and then we can get the following equation:

(14) $$\{\begin{array}{c} m_{i}=\frac{\partial g_{i}(x,y)}{\partial x}{|}_{\left(x,y\right)}=(x^{\prime },y^{\prime })=\frac{x^{\prime }-b_{i}}{\sqrt{{\left(x^{\prime }-a_{i}\right)}^{2}+{\left(y^{\prime }-b_{i}\right)}^{2}}}\\ n_{i}=\frac{\partial g_{i}(x,y)}{\partial y}{|}_{\left(x,y\right)}=(x^{\prime },y^{\prime })=\frac{y^{\prime }-b_{i}}{\sqrt{{\left(x^{\prime }-a_{i}\right)}^{2}+{\left(y^{\prime }-b_{i}\right)}^{2}}}\\ g_{i}=g_{i}(x^{\prime },y^{\prime })=\sqrt{{\left(x^{\prime }-a_{i}\right)}^{2}+{\left(y^{\prime }-b_{i}\right)}^{2}}\\ g_{i}(x,y)\approx g_{i}+m_{i}(x-x^{\prime })+n_{i}(y-y^{\prime })=m_{i}x+n_{i}y+g_{i}-m_{i}x^{\prime }-n_{i}y^{\prime } \end{array}$$

Let $c_{i}=g_{i}-m_{i}x\prime -n_{i}y\prime -q_{i}$, $w_{1}=\frac{1}{\sigma_{1}^{2}}$, $w_{2}=\frac{1}{\sigma_{2}^{2}}$, we can get the following formula:

(15) $$f\left(x,y\right)=\overset{4}{\underset{i=1}{\sum }}f_{i}\left(x,y\right)=-\frac{1}{2}\overset{4}{\underset{i=1}{\sum }}\left\{w_{1}\left[{\left(x-x_{k-1}\right)}^{2}+{\left(y-y_{k-1}\right)}^{2}\right]+w_{2}{\left(m_{i}x+n_{i}y+c_{i}\right)}^{2}\right\}$$

(4) Calculate the target tag’s position. What makes p(x,y) to be the maximum value can be considered as the target tag’s location. Let the first derivative of Equation (15) equals zero, we can get the following equation:

(16) $$\{\begin{array}{c} (4w_{1}+w_{2}\sum_{i=1}^{4}m_{i}{}^{2})x+w_{2}(\sum_{i=1}^{4}n_{i}m_{i})y+w_{2}\sum_{i=1}^{4}c_{i}m_{i}-4w_{1}x_{k-1}=0\\ w_{2}(\sum_{i=1}^{4}n_{i}m_{i})x+(4w_{1}+w_{2}\sum_{i=1}^{4}n_{i}{}^{2})y+w_{2}\sum_{i=1}^{4}c_{i}n_{i}-4w_{1}y_{k-1}=0 \end{array}$$

Then the value of (x,y) which is the position of the target tag can be calculated using Equation (16).

## 5. Experiments and Results

### 5.1. Experimental Settings

**Figure 6:**
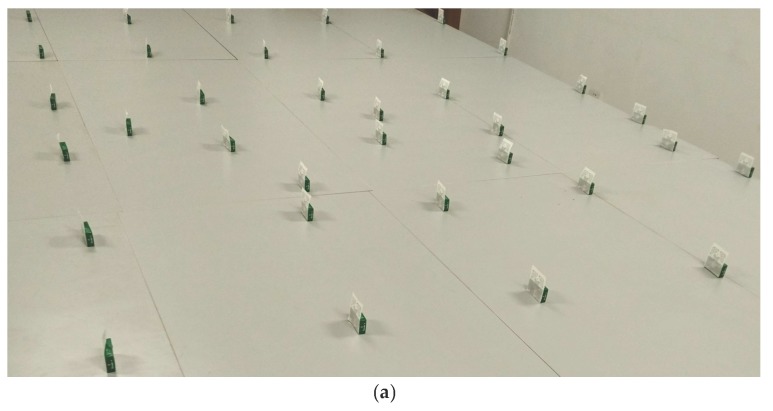

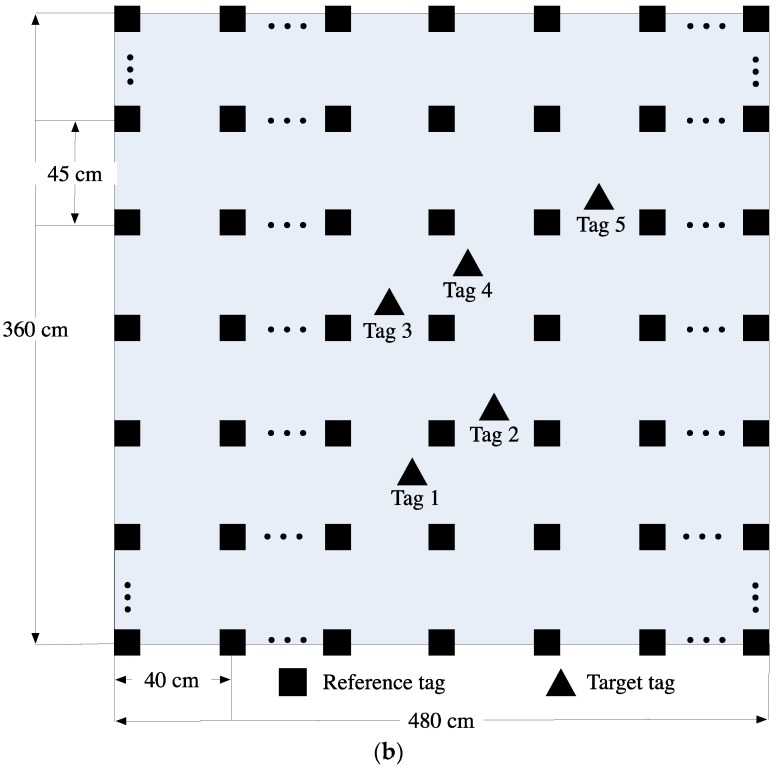
Experimental environment. (**a**) part of the monitoring area; (**b**) every tag space duration distance.

Figure 6 shows the experimental environment. In our experiments, Impinj R420 RFID reader and EPC Gen2 tags are used. The experimental settings are listed in Table 2. In the experiments, the whole test area is 3.6 m × 4.8 m. We placed 117 reference tags positioned in 9 rows by 13 columns in the monitoring area of the RFID reader (Figure 6a shows part of the monitoring area) and 5 target tags which needed to be positioned. The matchboxes were used to bolster tags in the experiments. The detailed experimental settings with every tag space duration distance are shown in Figure 6b, where the squares and triangles represent reference tags and to-be-positioned target tags, respectively.

### 5.2. Results of the Gaussian Filter for RSS

**Figure 7:**
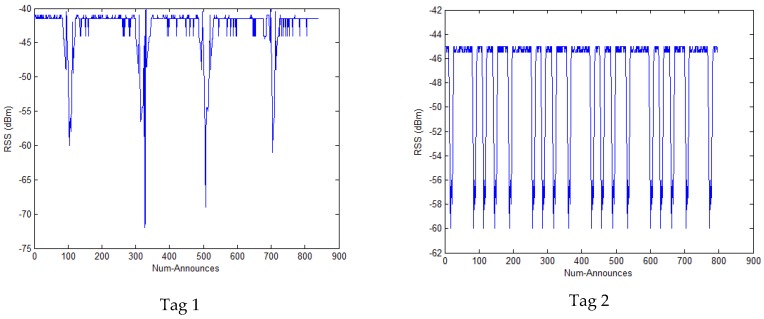

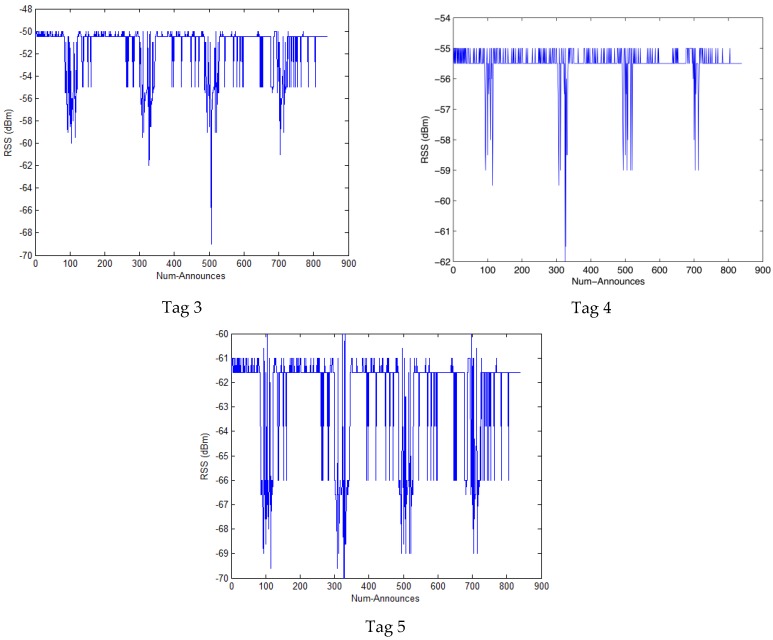
The RSS values of 5 tags.

**Figure 8:**
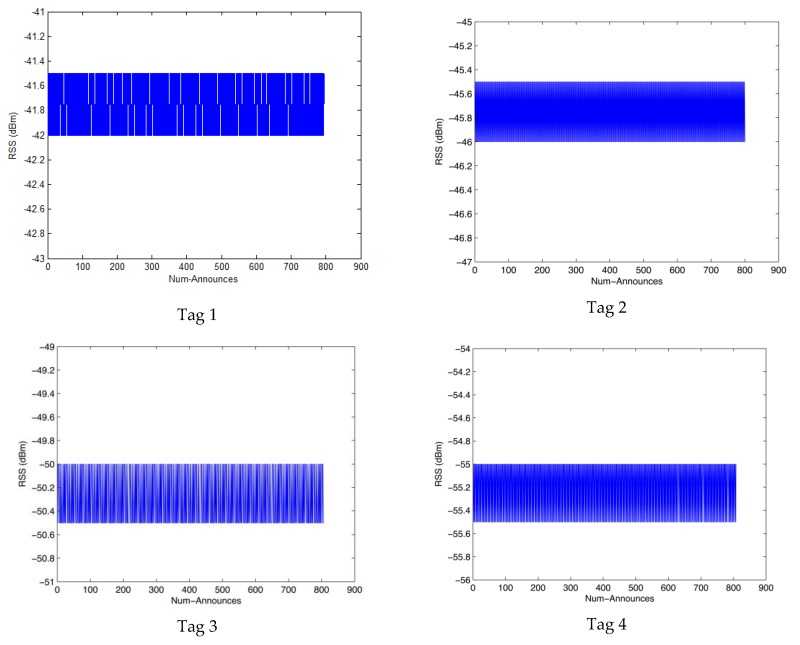

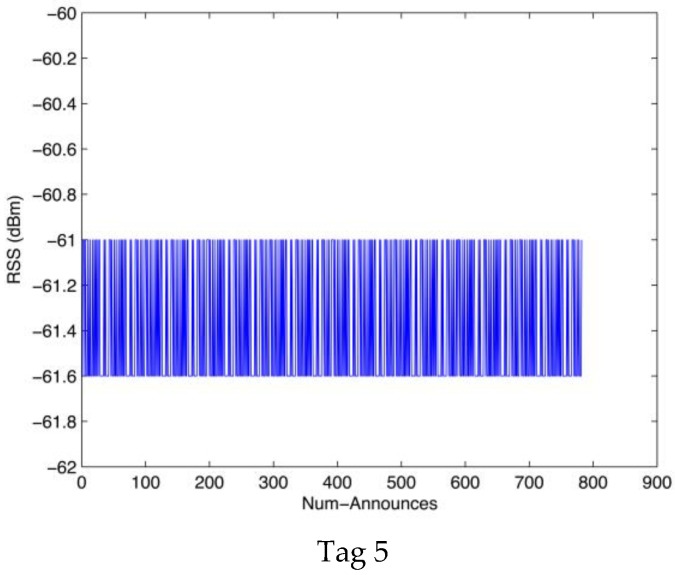
The RSS value of 5 tags processed by the Gaussian filter.

We collected the 5 tags’ RSS values using the R420 RFID reader, Figure 7 shows the results of the RSS values where we can see that there are many fluctuations. The Gaussian filter is used to process these abnormal data, Figure 8 shows the results of the filtered RSS value. We can see that the RSS values processed by the Gaussian filter are stable to some extent and there are no large fluctuations.

### 5.3. Results of the Average Location Error

**Figure 9:**
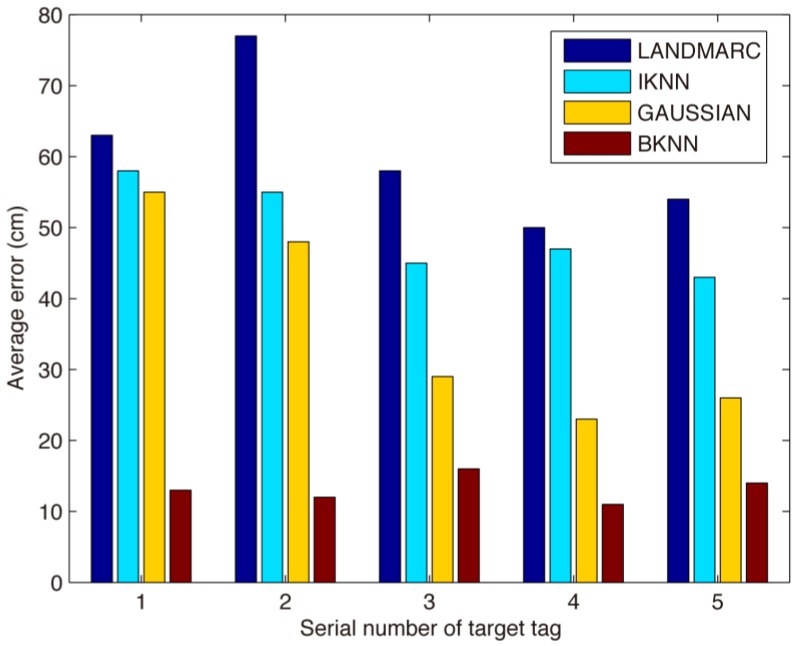
The average error of location estimation of 5 tags.

Firstly, we selected the original data obtained from the RFID reader and used the LANDMARC algorithm to position unknown tags. Then the average location error was compared among the LANDMARC algorithm [25], the IKNN algorithm [43], the Gaussian-based algorithm and our BKNN algorithm. In the experiments, 4 reference tags are selected to locate target. We performed 10 timed tests, and each test took about 20 min. So the sum of Num-Announces of each selected reference tag was about 8000, and all of those RSS values were collected and used by positioning algorithms to estimate the 5 target tags. Figure 9 shows the results of the average location error among the compared algorithms. We can see that the location error of the Gaussian-based algorithm is less than that of the LANDMARC and IKNN algorithm, this is because some abnormal RSS values can be filtered by the Gaussian filter. The average error of the BKNN location estimation is about 15 cm, which is the smallest among the compared algorithms. This is because our BKNN uses the proper k value and processed RSS values which can remove abnormal location data, and uses Bayesian probability to estimate the target tag’s location which can predict the exact location data. Thus, BKNN reduces the location error and has a much higher location accuracy than that of other compared algorithms. According to literature [43], the tag space between the two reference tags is 1 m, but the average error of location estimation is about 85 cm, while the average error of our algorithm is about 15 cm when the tag space is 40 cm. Though we use more reference tags, the accuracy is improved, which has significant implications for practical indoor positioning algorithms. In low cost RFID-based indoor positioning environments, the average accuracy of the location estimation of our algorithm is also very effective and suitable for use in indoor location based services, i.e., book searching in the library, locating patients in a hospital, finding people trapped in a burning building, finding an individual’s luggage without removing it from the vehicle or the aircraft in an airport and reminding passengers of flights and boarding gates so that they do not miss their flights or delay departures.

### 5.4. Comparisons of BKNN with Different Positioning Methods

In this section, for precision, cost, time consumption, energy consumption, etc., we compare BKNN with other positioning methods, such as LANDMARC [25], fingerprint-based [33], phase-based [34] and IKNN [43]. Table 3 shows the results of the comparisons. As passive UHF RFID was used, the cost and energy consumption is low in our BKNN algorithm. All the compared algorithms use the COTS RFID reader which can easily be implemented in current RFID systems, and because the abnormal data is filtered before calculating the final location, the time consumption is lower than that of the IKNN and LANDMARC algorithms.

## 6. Conclusions

This paper has presented an indoor positioning method using passive UHF RFID. However, there are two problems that passive RFID encounters when used in an indoor location. The first problem is that the RSS value of RFID is easily affected by the surrounding dynamic environment. The second problem is the phase value is cyclical and the phase difference is tag sensitive, which makes UHF RFID difficult to use in large-scale locations. In this paper, we mainly solve the noise problem which influences the location accuracy. A Gaussian filter is used to filter abnormal RSS values and Bayesian estimation together with the KNN algorithm are used to improve positioning accuracy. In the experiments, 4 reference tags are selected to locate target. The average location error of our algorithm is about 15 cm, which is the smallest among the compared algorithms. Because the positioning accuracy has a relationship with the collected RSS values of reference tags, how to deploy the optimized placement of readers and reference tags will be studied in our future work.

## Figures and Tables

**Table 1:** Comparisons of different positioning methods.

	Index	Accuracy	Time Consumption	Cost	Energy Consumption	COTS Reader
Positioning Methods						
Fingerprint [10,33]		High	Low	High	Low	Yes
Reference tags [25]		Normal	High	Normal	Normal	Yes
AOA [31]		High	Normal	Normal	High	Yes
TDOA [6]		Low	Low	Normal	Low	No

**Table 2:** The experimental settings.

Parameter	Value
Range	3.6 m × 4.8 m
Numbers of reference tags	117
Numbers of target tags	5

**Table 3:** Comparisons of BKNN with different positioning methods.

	Index	Precision	Time Consumption	Cost	Energy Consumption	COTS Reader
Positioning Methods						
LANDMARC [25]	Normal	High	Normal	Normal	Yes
Fingerprint [33]	High	Low	High	Low	Yes
Phase [34]	High	High	Normal	Normal	Yes
IKNN [43]	High	High	Normal	Normal	Yes
Proposed BKNN	High	Normal	Low	Low	Yes
